# The need for surgery in pediatric patients with inflammatory bowel disease treated with biologicals

**DOI:** 10.1007/s00384-024-04634-7

**Published:** 2024-04-25

**Authors:** Kaija-Leena Kolho, Anne Nikkonen, Laura Merras-Salmio, Pauliina Molander

**Affiliations:** 1 Children’s Hospital, Helsinki University Hospital HUS and Helsinki University, Helsinki, Finland; 2 Abdominal Center, Gastroenterology, Helsinki University Hospital HUS and Helsinki University, Helsinki, Finland

**Keywords:** Anti-TNFα, Children, Crohn´s disease, Infliximab, Ulcerative colitis

## Abstract

**Purpose:**

Inflammatory bowel disease (IBD) in childhood often presents with a more extensive and more aggressive disease course than adult-onset disease. We aimed to evaluate if biological treatment started in childhood decreases the need for intestinal surgery over time.

**Methods:**

This was a retrospective, single-center, cohort study. All pediatric patients with IBD initiated to biological therapy at the Children’s Hospital, were included in the study and followed up to the first surgical procedure or re-operation in their adulthood or until 31.12.2021 when ≥ 18 of age. Data were collected from the pediatric registry of IBD patients with biologicals and medical charts.

**Results:**

A total of 207 pediatric IBD patients were identified [150 with Crohn´s disease (CD), 31 with ulcerative colitis (UC), 26 with IBD unclassified (IBDU)] of which 32.9% (*n* = 68; CD 49, UC 13, IBDU 6) underwent intestinal surgery. At the end of a median follow-up of 9.0 years (range 2.0-25.9), patients reached a median age of 21.4 years (range 18–36). Patients who had intestinal surgery in childhood were more likely to have IBD-related surgery also in early adulthood. The duration of the disease at induction of the first biological treatment emerged as the only risk factor, with a longer duration in the surgical group than in patients with no surgery.

**Conclusion:**

Despite initiation of biological treatment, the risk of intestinal surgery remains high in pediatric IBD patients and often the need for surgery emerges after the transition to adult IBD clinics.

## Introduction

Pediatric inflammatory bowel disease (PIBD) often presents with more extensive disease especially in UC, and a more aggressive disease course than adult-onset disease [1]. One study reported a 90% frequency of pancolitis in children diagnosed with UC in contrast to 37% of those diagnosed in adulthood [2]. Moreover, up to 20–35% of patients with colonic disease are classified as IBD–unclassified (IBDU), with clinical and endoscopic signs of chronic colitis without specific features of UC or CD but features of both, which may lead to difficulties in choosing the most suitable medical therapy or surgical intervention [3]. In addition, children with pre-pubertal onset of IBD and those with a more severe disease course are at risk for hospitalization and surgery [4].

Despite significant earlier use of immunomodulators and biological therapy in recent years, the need for surgery remains necessary to relieve symptoms and treat disease complications [3]. As surgery in CD is not curative, the principle is to undertake a limited bowel resection, thus preserving bowel length for the future. In CD surgical options vary from single-site resection (most commonly ileocaecal resection) to subtotal colectomy and the laparoscopic approach is considered the gold standard [5]. In UC, colectomy may involve 1–3 stages with initial subtotal colectomy and ileostomy with oversewn rectal stump. In the next stage, restorative procto-colectomy with removal of the remaining rectum and either ileo-anal anastomosis (IAA) or an ileal pouch-anal anastomosis (IPAA) is performed. If this second procedure is performed with protective ileostomy remaining, the third stage procedure is seclusion of the stoma. Several studies have shown that children with IBD have relatively low risks of surgery or intestinal complication; the 1-year cumulative risk of surgery in the pediatric population ranges from 2.2 to 7% in CD and 2.4 to 8% in UC, and from 10 to 30% in all pediatric IBD patients within 5 years of diagnosis [3, 4, 6–12]. However, the lifetime risk of surgery for pediatric UC ranges from 15 to 45% and is up to 80% for CD and up to 30% of them will undergo repeated surgeries due to recurrences [13–15]. Only one study showed the reduction of surgical resections during childhood for PIBD during the same timeframe as the use of biological therapy increased [16]. It is not clear whether the requirement for surgical intervention is only delayed, or whether surgery is completely avoided with biological therapy. We aimed to evaluate if biological treatment started in childhood decreases the need for intestinal surgery in early adulthood.

## Materials and methods

### Study design

The study cohort included all patients with a diagnosis of CD (K50; The International Classification of Diseases, 10th revision) or UC/IBDU (K51) who initiated biologicals before turning 18 years of age at the Children’s Hospital, University of Helsinki, Finland. Infliximab, as the first biological treatment indicated for the treatment of pediatric IBD, has been available in Finland since 1999. The data were collected retrospectively from the patient charts and the Institutional IBD biological registry, which has been in routine use and includes all patients initiated to biological therapy at the Children’s Hospital. We included patients who had reached a minimum of 18 years at the end of the data collection. Patients were followed up until 31.12.2021 or until their first surgical procedure in adulthood (age ≥ 18) whichever occurred first.

We collected data on patient age at diagnosis and sex, localization, and behavior of PIBD, medication used, family history, concomitant diseases, age, and the disease duration at the induction of the first biologic and the length of follow-up. In addition, we reviewed the data on growth delay at diagnosis and pubertal delay during follow-up.

The physicians’ global assessment (PGA) score was used to determine disease activity at diagnosis. As no validated PGA score exists, PGA scores here were captured as follows: 1 = no or minor symptoms; 2 = occasional diarrhea, abdominal pain, or rectal bleeding indicating moderate disease; 3 = diarrhea several times a day/at night, or daily rectal bleeding indicating severe IBD [17].

### Statistical analyses

Baseline demographic characteristics of patients were compared between patients undergoing surgery and those treated conservatively. Values are expressed as a median and range unless otherwise stated. Continuous variables between the groups were analyzed using either the Mann-Whitney test or the t-test. All analyses were conducted using a two-tailed approach, and statistical significance was defined as *P*-values less than 0.05. Kaplan-Meier survival analysis served for the estimation of surgical-free survival rates using the log-rank test. Statistical analyses were conducted using SPSS (Statistical Package for the Social Sciences) Statistics 25 for Windows software (IBM Company, Chicago, IL, USA). Graphs have been created using GraphPad Prism version 9.4.1 (GraphPad Software, La Jolla, California, USA).

### Checklist for the reporting of observational studies

This article was written in adherence with the Strengthening the Reporting of Observational Studies in Epidemiology (STROBE) checklist for the reporting of observational studies [18].

### Ethical statement

This was a register-based study and, according to Finnish legislation, ethical approval or informed consent was not needed as the patients were not contacted.

## Results

### Patient characteristics

The study cohort consisted of 207 patients with pediatric-onset IBD including 150 CD, 31 UC, and 26 IBDU patients treated with biologicals, and followed up for a median of 9.0 years (range 2.0–25.9) until a median age of 21.4 years (range 18–36) at the end of follow-up. The subgroups of UC and IBDU are herein united in the UC/IBDU group. A slightly higher proportion of patients were male (58.9%). According to the Paris classification, most CD patients had either colonic or ileocolonic disease with or without upper gastrointestinal (GI) disease (L2 26.7%; L3 27.3%; L2+L4b 12.0%, L3+L4b 21.3%) and the predominant behavioral phenotype was non-stricturing, non-penetrating (B1, 79%), followed by non-stricturing, non-penetrating with perianal disease (B1p, 15%) disease. Most UC and IBDU patients had pancolitis (75.4%). The mean PGA at diagnosis was 2 (range 1–3). Nearly half of the patients (*n* = 89, 43%) had at least one extraintestinal manifestation. The most common extraintestinal manifestation was orofacial granulomatosis (*n* = 18, 12.1%, all CD) followed by primary sclerosing cholangitis (PSC) with or without features of autoimmune hepatitis (AIH) (*n* = 14, 6.8%). Expectedly, 202 (97.6%) patients had a TNF alpha inhibitor (infliximab *n* = 196, adalimumab *n* = 4, golimumab *n* = 2) as a first-line biological treatment and the median age at initiation of the first biological treatment was 14.9 years. Patient characteristics are shown in Table 1.

**Table 1:** Demographic and clinical data of the cohort

**Total number of patients,** ***n*** **= 207**	**CD,** ***n*** **= 150**	**UC,** ***n*** **= 31**	**IBDU,** ***n*** **= 26**
**Sex, male (%)**	102 (68)	10 (32)	10 (39)
**Age at diagnosis, median (range)**	13.3 (2–16.5)	12.3 (4–16)	13.8 (3–16)
**PGA at diagnosis (scale 1–3), mean (*n*)**	1.8 (128)	1.8 (29)	2.3 (24)
**Age at diagnosis, Paris classification,** ***n*** **(%)**			
** A1a (0 to < 10y)**	29 (19.5)	6 (19)	3 (11.5)
** A1b (10 to < 17y)**	121 (80.5)	25 (81)	23 (88.5)
** A2 (17–40y)**	0	0	0
**Paris classification for CD,** ***n*** **(%)**			
** Location**			
** L1**	7 (4.7)		
** L1+L4b**	4 (2.7)		
** L4b**	6 (4)		
** L2**	40 (26.7)		
** L2+L4b**	18 (12)		
** L3**	41 (27.3)		
** L3+L4b**	32 (21.3)		
** Perianal only**	2 (1.3)		
** L4a**	38 (25.3)		
** Behavior**			
** B1**	118 (79)		
** B1 p**	23 (15)		
** B2**	8 (5)		
** B2 p**	1 (1)		
**Paris classification for UC/IBDU,** ***n*** **(%)**			
** E1**		0	0
** E2**		6 (19.4)	6 (23.1)
** E3**		1 (3.2)	1 (3.8)
** E4**		24 (77.4)	19 (73.1)
**Growth,** ***n*** **(%)**			
** G0**	115 (76.7)	30 (96.8)	25 (96.2)
** G1**	35 (23.3)	1 (3.2)	1 (3.8)
**Pubertal delay,** ***n*** **(%)**	21 (15.3)	3 (9.7)	0
**Concomitant diseases,** ***n*** **(%)**			
** Orofacial granulomatosis**	18 (12.1)	0	0
** Rheumatoid arthritis**	2 (1.3)	0	3 (11.5)
** PSC ± AIH features**	8 (5.4)	5 (16.1)	1 (3.8)
** Diabetes mellitus**	2 (1.3)	0	0
** Celiac disease**	3 (2.0)	0	3 (11.5)
** IgA nephropathy/nephritis/megaureter**	7 (4.7)	1 (3.2)	1 (3.8)
** Psoriasis**	7 (4.7)	2 (6.5)	0
** Asthma**	34 (22.7)	7 (22.6)	2 (7.7)
** Atopic dermatitis**	46 (30.7)	4 (12.9)	7 (26.9)
** Hidradenitis suppurativa**	1 (0.7)		
** Other**	16 (10.7)	4 (12.9)	4 (15.4)
**Treatments prior to introduction of biologicals,** ***n*** **(%)**			
** Corticosteroids**	132 (88)	31 (100)	26 (100)
** 5-ASA/Sulphasalazine**	121 (80.7)	31 (100)	25 (96)
** Azathioprine/6-Mercaptopurine**	100 (66.7)	29 (94)	23 (89)
** Methotrexate**	38 (25.3)	9 (29)	8 (31)
** Cyclosporine**	3 (2)	2 (7)	1 (4)
** Exclusive enteral nutrition**	18 (12)		
**First line biological treatment,** ***n*** **(%)**			
** anti-TNFα agents**	150 (100)	29 (93.5)	23 (88)
** Vedolizumab**	0	2 (6.5)	3 (12)
**Number of biological agents used at the end of follow up,** ***n*** **(%)**			
** 1**	82 (54.7)	14 (45)	13 (50)
** 2**	41 (27.3)	11 (35.5)	9 (35)
** 3**	16 (11)	4 (13)	4 (15)
** 4**	7 (4.7)	2 (6.5)	
** 5**	4 (2.7)		

*AIH* Autoimmune hepatitis, *5-ASA* 5-aminosalicylic acid, *CD* Crohn´s disease, *IBDU* Inflammatory bowel disease unclassified, *PGA* Physician´s global assessment, *PSC* Primary sclerosing cholangitis, *TNF* Tumour necrosis factor, *UC* Ulcerative colitis

### Intestinal surgery

**Fig. 1:**
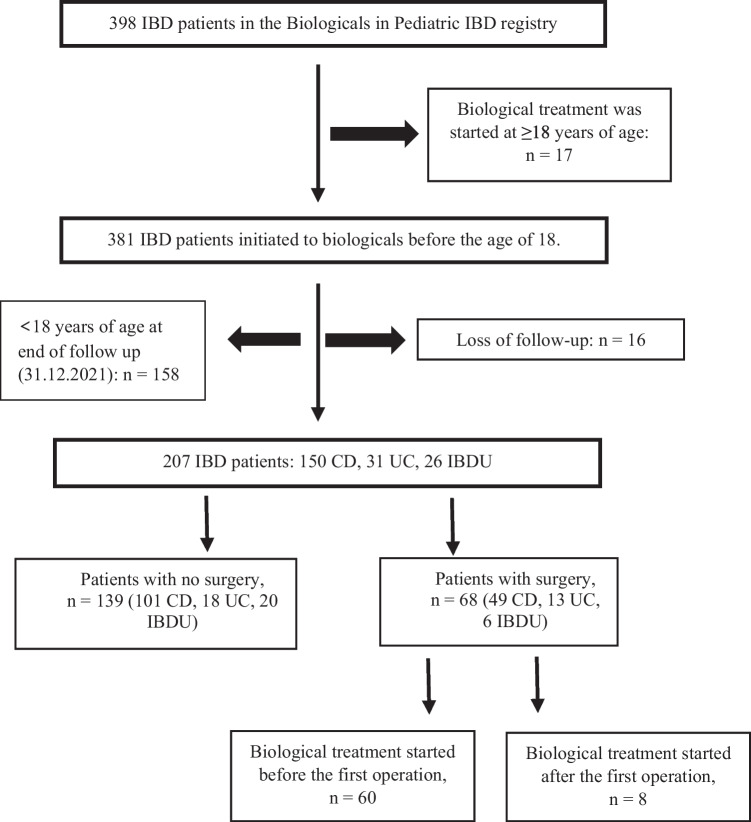
The flow chart of patient enrollment. CD = Crohn´s disease; IBD = inflammatory bowel disease unclassified; IBDU = inflammatory bowel disease unclassified; UC = ulcerative colitis

Altogether 68 patients (32.8%; CD 49/150, UC/IBDU 19/57) underwent intestinal surgery, because of inadequate response to medical therapy, Fig. 1. The characteristics of the patients in the surgical group compared to those without IBD-related surgery were mostly identical, Table 2. However, the duration of the disease at induction of the first biological treatment was significantly longer in the surgery group compared to patients who did not undergo surgery (1.9 vs. 1.0 years, *p* = 0.020). In the surgical group, 11.9% of patients (*n* = 8, all CD) were initiated to biological therapy within 3 months after the diagnosis compared to 24.1% (CD 33, IBDU 1) without IBD-related surgery (*p* = 0.042). The median age at the first operation was 16.9 years (range 10.3–31.3). In the surgical group, 46 patients (67.6%; CD 33, UC/IBDU 13) were under 18 years of age at the time of the first surgery, and 22 patients (32.4%; CD 16, UC 5, IBDU 1) were older. Five patients were diagnosed with VEO-IBD (diagnosis < 6 years of age, all with IBDU), and only one of these patients had surgery (at the age of 11). Colectomies and proctocolectomies were performed as one-, two-, or three-stage operations. In the group of UC/IBDU patients having colectomies under the age of 18 (*n* = 13), six patients had two-stage surgery and seven had three-stage surgery. In contrast, of the older patients undergoing colectomies (18 years of age or older, *n* = 6), three had one-stage surgery, while the other three had two-stage surgery and none had three-stage surgery. One patient with CD underwent bowel resection after sigma perforation caused by endoscope at the age of 15 and had subtotal colectomy because of an inflammatory stricture in the sigmoid colon later in the same year. Types of the first IBD-related surgical procedures in all patients are presented in Table 3.

**Table 2:** Patient characteristics based on the need for surgery

**Total number of patients** ***n*** **= 207**	**Patients with no surgery,** ***n*** **= 139**	**Patients with surgery,** ***n*** **= 68**	***p*** **value**
**IBD diagnosis,** ***n*** **(%)**			
** Crohn’s disease (CD)**	101 (73)	49 (72)	
** Ulcerative colitis (UC)**	18 (13)	13 (19)	
** IBD unclassified**	20 (14)	6 (9)	
**Sex, male (%)**	85 (61)	37 (54)	
**Age at diagnosis, median (range)**	13.6 (2.1–16.5)	13.3 (5.1–16)	*p* = 0.552
**PGA at diagnosis, scale 1–3, mean (*n*)**	1.9 (126)	1.8 (56)	*p* = 0.379
**Length of follow up, years, median (range)**	8.2 (2.2–25.9)	9.5 (2.3–24.5)	*p* = 0.780
**Age at diagnosis, Paris classification,** ***n*** **(%)**			
** A1a (0 to < 10y)**	18 (13)	20 (29.4)	
** A1b (10 to < 17y)**	121 (87)	48 (70.6)	
**Paris classification for CD,** ***n*** **(%)**			
** Location at diagnosis**			
** L1**	4 (4)	3 (6.1)	
** L1+L4b**	2 (2)	2 (4.1)	
** L4b**	4 (4)	2 (4.1)	
** L2**	24 (23.7)	16 (32.7)	
** L2+L4b**	15 (14.9)	3 (6.1)	
** L3**	24 (23.7	17 (34.7)	
** L3+L4b**	26 (25.7)	6 (12.2)	
** Perianal only**	2 (2)	0	
** L4a**	26 (25.7)	12 (24.5)	
** Behavior at diagnosis**			
** B1**	97 (96)	44 (89.8)	
** B2**	4 (4)	5 (10.2)	
** + Perianal manifestation at diagnosis**	16 (15.8)	8 (16.3)	
**Paris classification for UC/IBDU,** ***n*** **(%)**			
** E1**	0	0	
** E2**	7 (18.4)	5 (26.3)	
** E3**	2 (5.3)	0	
** E4**	29 (76.3)	14 (73.7)	
**Growth delay at diagnosis,** ***n*** **(%)**	23 (16.6)	14 (28.6)	
**Pubertal delay during follow-up,** ***n*** **(%)**	14 (10.1) Missing data, *n* = 23	10 (20.4) Missing data, *n* = 16	
**Family history,** ***n*** **(%)**	*n* = 107	*n* = 48	
** 1st Degree (parents, siblings) relatives**	15 (14)	9 (18.7)	
** Other relatives**	19 (17.8)	11 (22.9)	
** Both 1st Degree and other relatives**	3 (2.8)	1 (2.1)	
**Concomitant diseases**			
** Orofacial granulomatosis**	15 (10.8)	3 (4.4)	
** PSC ± AIH features**	8 (5.8)	6(8.8)	
** Rheumatoid arthritis**	4 (2.9)	1 (1.5)	
** Diabetes**	2 (1.4)	0	
** Celiac disease**	4 (2.9)	2 (2.9)	
** IgA nephropathy/nephritis/megaureter**	5 (3.6)	3 (4.4)	
** Psoriasis**	7 (5)	1 (1.5)	
** Asthma**	26 (18.7)	17 (25)	
** Atopic dermatitis**	38 (27.3)	19 (27.9)	
** Hidradenitis suppurativa**	1 (0.7)	0	
** Other**	12 (8.6)	14 (20.6)	
**Age at the time of initiation of the first biologic agent, years, median (range)**	14.9 (2.8–17.9)	14.9 (6.5–17.7)	*p* = 0.799
**Disease duration at the time of start of first biologic agent, years, median (range)**	1.0 (0–8.4)	1.9 (0–8.3)	*p* = 0.020
**First line biological treatment,** ***n*** **(%)**			
** anti-TNFα agents**	130 (97)	61 (98.5)	
** Vedolizumab**	4 (3)	1 (1.5)	
**IBD-associated hospitalization (other than surgery related),** ***n*** **(%)**	69 (49.6)	51 (76.1) Missing data, *n* = 1	*p* = 0.544

Significance level is 0.05

*AIH* Autoimmune hepatitis, *CD* Crohn´s disease, *IBDU* Inflammatory bowel disease unclassified, *PGA* Physician´s global assessment, *PSC* Primary sclerosing cholangitis, *TNF* Tumour necrosis factor, *UC* Ulcerative colitis

**Table 3:** Types of the first intestinal surgery

**Procedures**	**CD patients** **(*n*** **= 49)**	**UC patients** **(*n*** **= 13)**	**IBDU patients** **(*n*** **= 6)**
Ileocaecal resection	18		
Proctocolectomy	7		
IPAA with J-pouch		9	2
Colectomy	4		
Colectomy and ileostomy		4	4
Colectomy + small bowel resection	2		
Hemicolectomy (left)	6		
Sigmoid resection	1		
Small bowel resection	7		
Ileostomy	2		
Ileorectal anastomosis	2		

*CD* Crohn´s disease, *IBDU* Inflammatory bowel disease unclassified, *IPAA* Ileal pouch-anal anastomosis, *UC* Ulcerative colitis

As in the whole cohort, the most common biological treatment in the group with surgery was infliximab (*n* = 66, 97.0%). Notably, eight CD patients (11.8%) were introduced to biological treatment after their first operation in childhood (ileum resection *n* = 4, ileocaecal resection *n* = 2, hemicolectomy *n* = 2). In this group of patients, the median time from the operation to the initiation of biological treatment was 5 months (range 1–68). Trough levels of infliximab and anti-drug antibodies were routinely available at the Children’s Hospital before every infliximab infusion since August 2010. Three-fourths (74.5%, CD 36, UC/IBDU 14) of patients undergoing surgery had trough level measurements available prior to surgery. Only one patient having surgery in childhood had a low infliximab level (0.46 µg/ml), and developed antibodies after surgery, and hence was switched to adalimumab postoperatively. In addition, one patient having surgery in adulthood had an undetectable level of infliximab before the operation, but no antibodies were reported.

### Reoperations

**Fig. 2:**
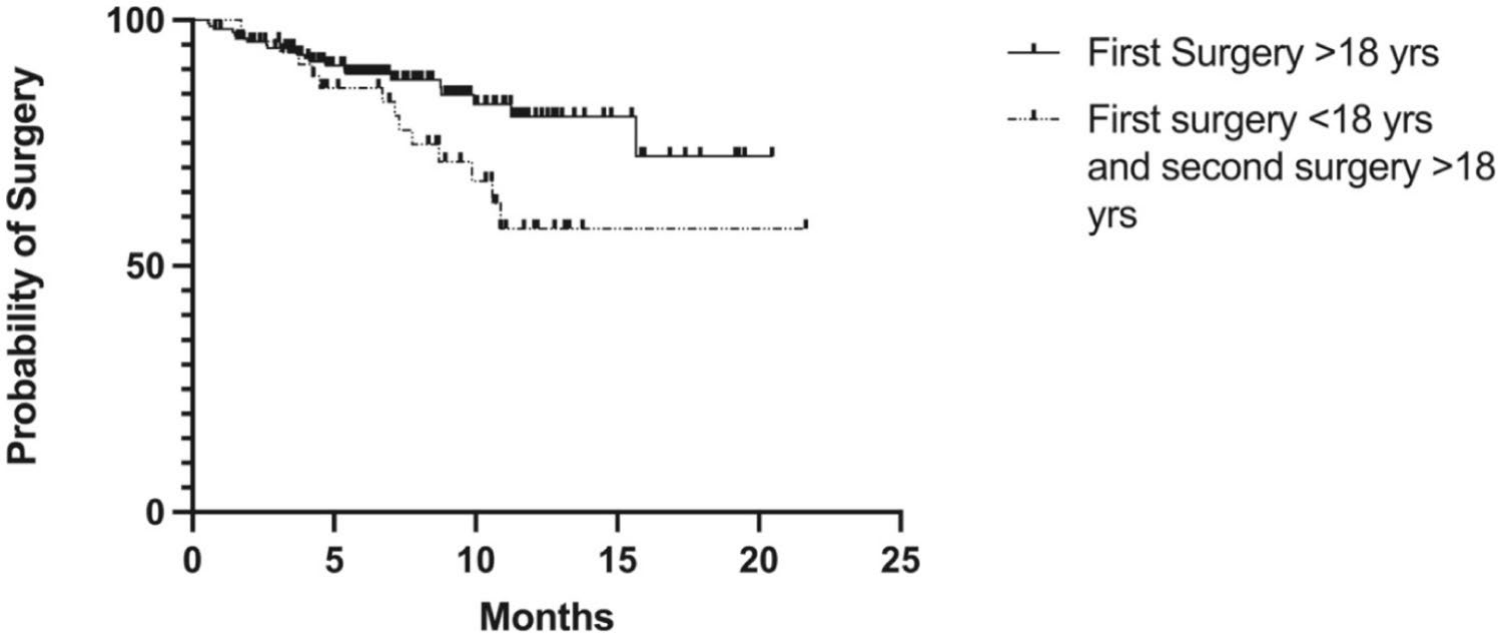
Biological therapy in pediatric-onset inflammatory bowel disease (PIBD) and risk for surgery in adulthood. Cumulative probability of surgery for inflammatory bowel disease (IBD) in adulthood in patients with PIBD treated with biologics and needing first surgery already during childhood compared to those with no surgery during childhood. *P = 0.0340* (log-rank test) for the difference between the curves

Nearly half of patients having surgery in childhood underwent a re-operation (*n* = 18/46, 39.1%) during the follow-up time. In detail, five patients (11%, CD 4, IBDU 1) underwent reoperations in childhood at the median age of 15.1 (13.5–16.3) and 13 patients in adulthood (28.3%; CD 11, IBDU 2) at a median age of 19.7 (18–24.9). Reoperations included bowel resections and ileal re-anastomosis, colectomies, one permanent ileostomy, one revision of the anal canal, and proctectomies because of active disease. Importantly, when comparing the cumulative probability of IBD-related surgery in PIBD patients treated with biologics and needing first surgery already during childhood to those with no intestinal surgery during childhood, the risk of surgery was significantly increased, *p* = 0.0340, Fig. 2. A statistical difference was found in the mean number of different biological treatments used during the follow-up time in patients having reoperation in adulthood compared to patients not being reoperated (3.33 (1–5) vs. 2.48 (1–6), *p* = 0.044). Moreover, eight patients (36.4%, CD 7, UC 1) who had their first surgery at the age of 18 or older were reoperated after a median follow-up of 2 years (0–10). One patient with CD and ileocecal resection needed surgery for adenocarcinoma later in adulthood.

## Discussion

In this real-life, retrospective, observational study we demonstrated that despite biological treatment initiated in childhood, the rate of surgery remains high and often the need for reoperation emerges after the transition to adult IBD clinics. However, the initiation of biological treatment less than 3 months from the diagnosis of PIBD seems to reduce the risk of surgery in CD.

Treatment goals in PIBD patients include achieving intestinal healing, reaching growth potential, and optimizing the quality of life, all while limiting drug toxicities [19]. In the last two decades, the introduction of TNFα-agents has significantly increased the potential to reach these goals. Like in the adult population, biologic therapy is indicated as induction and maintenance therapy in situations in which the patient with PIBD has not responded to previous therapies or when the step-up approach is not considered appropriate due to extensive and/or aggressive disease [19, 20]. However, nonresponse or loss of response to anti-TNFα agents is still encountered in approximately one-third of patients. Based on the REACH study (randomized, multicenter, open-label study), 55.8% (infliximab every 8 weeks) and 23.5% (infliximab every 12 weeks) of adult patients reached clinical remission [21]. Similar findings on efficacy and safety have been reported in an RCT of 60 children with moderate to severe UC receiving infliximab and in children with moderate to severe CD receiving adalimumab [22, 23]. In children, indications for surgery are comparable to adults, including inadequate response to medical therapy or unacceptable side-effects of medical therapy, toxic megacolon, complications of the disease (fistula, obstruction, perforation, abscess formation, and bleeding), inadequate nutrition, development of dysplasia and especially in UC steroid dependency. Furthermore, in children, the indications for surgery may also be growth failure or delayed puberty [19]. A significant number of patients still need surgery as a treatment option in active IBD, which is also seen in our study, as up to one-third of PIBD patients underwent intestinal surgery because of inadequate response to biological therapy.

In our study, biological treatment was started within 1.5. years (mean) from diagnosis and did not prevent pediatric patients from having intestinal surgery in their childhood or early adulthood. In addition, the risk of re-operation remained notably high. In an earlier study by Piekkala et al., out of 36 pediatric CD patients, who underwent a bowel resection during childhood between the years 1985 and 2008, 94% required either medical or surgical treatment for active disease at a median of 1.8 years after the first surgery [24]. The patient population comprised all CD patients undergoing surgery during that time. Very few patients (8%) had biological treatment before surgery, contrary to the present study. In addition, in a large Danish cohort of 115 CD patients, up to 39% required further resection after the first bowel resection [25]. These findings are in line with our study, as 42% of CD patients here needed further surgical treatment. The question remains whether the introduction of biological therapy earlier rather than later in the disease course would decrease surgical intervention for children. Walters et al. addressed this issue in a multicenter cohort of pediatric CD patients comparing anti-TNFα therapy (infliximab, adalimumab) and immunomodulators (thiopurines, methotrexate) administered within three months after diagnosis to no early immunomodulatory therapy [26]. By one year of follow-up, anti-TNFα therapy was significantly more effective in achieving corticosteroid and surgery-free remission than immunomodulator-based monotherapy or no early immunotherapy (85.3% vs. 60.3% vs. 54.4%), whereas no significant difference between early immunomodulator based monotherapy to no early immunotherapy was found. Moreover, Jongsma et al. showed first-line infliximab to be superior to conventional treatment in achieving short-term clinical and endoscopic remission, and those on infliximab had a greater likelihood of maintaining clinical remission after one year than those on azathioprine monotherapy [27]. It is reasonable to state that in PIBD patients who often present with an aggressive disease course, biological treatment should be offered very early in the disease course.

Our study demonstrates that only the duration of the disease at induction of biological treatment was a risk factor for worse treatment outcomes. In an earlier study with adult CD patients, upper GI tract involvement was identified as an independent predictor of CD-related intestinal surgery, while early utilization of thiopurine was an independent protective factor against surgery [28]. However, in a Danish adult cohort, postoperative azathioprine did not decrease the rate of recurrence after surgery [25]. Moreover, Shinagawa et al. reported quite recently, that preoperative smoking, perianal, and ileocolic disease were significant risk factors for reoperation in CD, while postoperative use of immunomodulators and anti-TNFα therapy significantly reduced the risk of CD-related reoperation [29]. Smoking has been shown to increase the risk for advanced and difficult-to-treat disease, the risk of penetrating intestinal complications, strictures or fistulae, and the need for surgical resections (first or second surgery). However, passive smoking exposure in childhood is no longer considered a risk factor for incident CD [30]. Information on smoking habits in our study population was very seldom recorded, and thus this data was scarce. However, in Finland, approximately 6% of adolescents report regular smoking [31]. In an open-label randomized trial including 31 patients with CD from the age of 12 to 65, early intervention with infliximab monotherapy (compared with no infliximab) prevented clinical, serological, and endoscopic CD recurrence following ileocolic resection [32].

Our study has some limitations. The major criticism is associated with the retrospective data collection. Moreover, due to the study setting, complete data on the variables were not available for all patients. It should also be taken into consideration that the therapeutic attitude has changed into more active management over the last years and this could have an impact on the reoperation rate. However, it is unlikely to have a major impact on the number of resections. The most important data on biological treatment and surgical procedures were comprehensive. The major strengths of this study are the exceptionally long follow-up time from childhood to adulthood and the joint electronic patient chart in both pediatric and adult clinics enabling the homogeneous data collection. We believe that this single-center study, with a large number of PIBD patients included, provides important knowledge on still existing high risk of intestinal surgery and reoperation.

In conclusion, our study shows that despite advanced medical therapy, the risk of intestinal surgery remains high in pediatric IBD patients, and a relatively high number of PIBD patients, especially those with CD, require surgical treatment in their early adulthood.

## Data Availability

No datasets were generated or analysed during the current study.
